# Management of Chemotherapy Induced Nausea and Vomiting in Patients on Multiday Cisplatin Based Combination Chemotherapy

**DOI:** 10.1155/2015/943618

**Published:** 2015-09-03

**Authors:** Praveen Ranganath, Lawrence Einhorn, Costantine Albany

**Affiliations:** Indiana University School of Medicine, Indianapolis, IN 46202, USA

## Abstract

Introduction of cisplatin based chemotherapy has revolutionized the treatment of germ cell tumors. A common side effect of multiday cisplatin chemotherapy is severe nausea and vomiting. Considerable progress has been made in the control of these side effects since the introduction of cisplatin based chemotherapy in the 1970s. Germ cell tumor which is a model for a curable neoplasm has also turned into an excellent testing ground to develop effective strategies to prevent chemotherapy induced nausea and vomiting (CINV) in multiday cisplatin based regimens. The use of combination of a 5-hydroxytryptamine (HT)_3_ receptor antagonist, a neurokinin-1 (NK_1_) antagonist, and dexamethasone has greatly improved our ability to prevent and control acute and delayed CINV. Mechanism and pattern of CINV with multiday chemotherapy may differ from those in single day chemotherapy and therefore efficacy of antiemetic drugs as observed in single day chemotherapy may not be applicable. There are only few randomized clinical trials with special emphasis on multiday chemotherapy. Further studies are essential to determine the efficacy, optimal dose, and duration of the newer agents and combinations in multiday cisplatin based chemotherapy.

## 1. Introduction

Germ cell tumors are rare cancers accounting for only 1% of all malignancies in American males. The introduction of cisplatin based combination chemotherapy revolutionized the treatment of germ cell tumors. The majority of patients with testicular cancer are cured with standard dose cisplatin based combination chemotherapy [1]. A common side effect of cisplatin based regimens is severe nausea and vomiting. Cisplatin in germ cell tumors is administered for five consecutive days and is appropriately categorized as highly emetogenic chemotherapy (HEC) with patients being vulnerable to nausea and vomiting on all five days. Median number of emetic episodes with cisplatin based regimens in the 1970s and the 1980s for germ cell tumors on day 1 was ten and decreasing on subsequent days [2]. These symptoms were significantly debilitating for patients. Considerable progress has been made in the control of nausea and vomiting from those early days but there is still paucity of data on antiemetic regimens for patients undergoing multiday cisplatin combination chemotherapy regimen. Germ cell tumor, which is a model for a curable neoplasm, has also turned into an excellent testing ground to develop effective strategies to prevent chemotherapy induced nausea and vomiting (CINV) in these regimens. Phases II and III randomized clinical trials focusing on multiday chemotherapy regimens are summarized in Table 1. The purpose of this paper is to review our current understanding of CINV in multiday cisplatin regimens and to evaluate clinical agents available for prevention and treatment of CINV as well as areas of future research.

## 2. Pathophysiology

It has been established that different pathways exist in the body that induce emesis, each relying on a set of different neurotransmitters, including serotonin, dopamine, histamine, and substance P. Receptors for these neurotransmitters are found in high numbers in the dorsal vagal complex, area postrema, and gastrointestinal tract [3]. Cisplatin damages the GI tract and causes calcium dependent exocytic release of 5-hydroxytryptamine (HT)_3_ from enterochromaffin cells in the GI mucosa. Released 5-HT_3_ binds to its receptors on the vagal afferent neurons and this binding activates the chemoreceptor trigger zone (CTZ) and vomiting center (VC). When CTZ is activated, it also releases various neurotransmitters which in turn stimulate the VC. Once activated, the VC modulates efferent transmission to respiratory, vasomotor, and salivary centers as well as to abdominal muscles, diaphragm, and esophagus, resulting in emesis [3, 4].

Substance P is a neurotransmitter of the tachykinin family and is widely located in the central and peripheral nervous system, including gut, the nucleus tractus solitarius (NTS), and the area postrema. It acts through the neurokinin-1 (NK_1_) pathway and has been implicated in the pathogenesis of emesis [5]. Preclinical studies have shown that NK_1_-receptor antagonists prevent emesis by acting centrally within the NTS [6]. Evaluation of NK_1_ receptor antagonists in animal emesis models also indicated that they were highly effective against the delayed phase of emesis caused by cisplatin [5, 6]. Dopamine receptors are also present in CTZ and dopamine antagonists like phenothiazines, butyrophenones, and metoclopramide have been effective in treating CINV, although restricted by their side effects.

## 3. Historical Perspective

Phenothiazines, introduced as antipsychotic medications, were the first group of drugs to demonstrate activity in chemotherapy induced vomiting and remained the mainstay of antiemetic therapy for almost 3 decades from their introduction in the 1950s. Standard agents included chlorpromazine hydrochloride and prochlorperazine which mainly acted as dopamine antagonists. Low doses were not effective while higher doses carried risk of extrapyramidal reactions, liver dysfunction, marrow aplasia, and excessive sedation. They were also ineffective if patient failed to respond to it in the first course of treatment [7].

Metoclopramide, a substituted benzamide, was used in Europe for decades for prevention of motion sickness but was considered ineffective against chemotherapy induced nausea. But in the 1980s, it was discovered that massive doses of the drug (2 mg/kg given before and after chemotherapy) helped to minimize nausea and vomiting in most patients treated with cisplatin. However, high doses of metoclopramide were associated with troublesome Parkinsonian symptoms which were somewhat dissipated with addition of diphenhydramine [8].

Steroids, particularly dexamethasone, were tried alone and in various combinations with older agents with good control of nausea and vomiting. In combination with metoclopramide and other agents like lorazepam, it was noted that 50–60% of patients experienced no emesis compared to around 20% with either of the agents alone [9]. CINV prophylaxis with combination of dexamethasone and metoclopramide was the mainstay of treatment in the 1980s till introduction of ondansetron. At Indiana University, metoclopramide or lorazepam in combination with dexamethasone before and after cisplatin for the first 2 days, followed by chlorpromazine on the other 3 days, was utilized with adequate antiemetic efficacy in patients undergoing multiday cisplatin based chemotherapy.

Based on observations that smoking marijuana helps to alleviate CINV, tetrahydrocannabinol as an antiemetic agent was evaluated by Sallan et al. Antiemetic effect was observed in 14 of 20 tetrahydrocannabinol courses and in 0 of 22 placebo courses. No patient had vomiting episodes while experiencing a subjective “high” [10]. Subsequently, in a double-blind prospective study, Einhorn et al. demonstrated superiority of nabilone, a synthetic cannabinoid, compared to prochlorperazine in patients undergoing multiday cisplatin chemotherapy. In patients treated with prochlorperazine, the mean number of emetic episodes on the first day of multiple-day cisplatin therapy was 10.3 compared with 7.05 on nabilone. It was however associated with significant dry mouth and dysphoria [11]. This has not been replicated in the era of 5-HT_3_ receptor antagonists. A study by Meiri et al. evaluating dronabinol, ondansetron, or combination demonstrated equivalent efficacy between all groups and did not show any benefit from addition of cannabinoids to a 5-HT_3_ receptor antagonist [12].

## 4. 5-HT**_**3**_** Receptor Antagonist

Just as cisplatin revolutionized the cure rate for germ cell tumors, ondansetron, the first 5-HT_3_ receptor antagonist, greatly mitigated the severe cisplatin-associated nausea and vomiting [13]. In 1990, Einhorn et al. piloted ondansetron 0.15 mg/kg intravenously for 3 doses in a phase II study with 35 patients receiving 4- to 5-day cisplatin based regimens. Ten patients (29%) had no vomiting or retching throughout the entire study period and 18 patients (51%) experienced two or fewer emetic episodes during the entire study period. Ondansetron was very well tolerated in all 35 patients [14]. A subsequent phase III study by Sledge Jr. et al. [15] evaluated 3 intravenous doses of ondansetron 0.15 mg/kg intravenously or metoclopramide 1 mg/kg in patients undergoing multiday cisplatin chemotherapy. Proportion of patients with no emetic episodes throughout the entire study period was higher in the ondansetron arm (30% versus 9%, *p* = 0.077). Significantly fewer antiemetic treatment failures (more than five emetic episodes or withdrawal from the study) occurred with patients given ondansetron (9%) than with those given metoclopramide (50%) during the entire study period (*p* = 0.002). Although ondansetron clearly demonstrated superiority over metoclopramide as a single agent, 70% of patients treated with ondansetron in this study experienced at least one emetic episode during the 5-day treatment period. Ondansetron was very efficacious on the first day of chemotherapy and its effect diminished over subsequent days.

Other 5-HT_3_ receptor antagonists, such as granisetron and dolasetron, were subsequently approved by the FDA and have demonstrated equivalent efficacy and toxicity relative to ondansetron [16]. A single blind prospective study by the granisetron study group evaluated efficacy of prophylactic intravenous granisetron versus alizapride (a substituted benzamide) with dexamethasone in patients receiving fractionated chemotherapy (cisplatin or ifosfamide) for 5 days. Granisetron was superior to the combination in preventing nausea and vomiting, 54% versus 43% complete responders, respectively, in the cisplatin group. Adverse events were also lower in the granisetron group [17]. A subsequent, double-blind, randomized, and crossover comparison of single daily intravenous doses of granisetron compared with three daily intravenous doses of ondansetron in 5-day fractionated chemotherapy demonstrated equal efficacy, safety, and patient preference, with both agents achieving good control of emetic symptoms with 5-day complete response rates of 44.0% with granisetron compared to 39.8% in the ondansetron arm [18].

Single agent 5-HT_3_ receptor antagonists were ineffective in prevention of delayed CINV, which is a major issue with multiday cisplatin based chemotherapy. A meta-analysis of 5 studies comparing a 5-HT_3_ receptor antagonist as monotherapy compared to placebo showed no clinical evidence of improvement of control of delayed emesis with addition of 5-HT_3_ receptor antagonists [19]. In early clinical trials, addition of dexamethasone consistently improved efficacy compared to 5-HT_3_ receptor antagonist alone, making it the standard for patients receiving cisplatin based therapy. In a multicenter trial looking at ondansetron plus dexamethasone compared to ondansetron alone in cisplatin based chemotherapy, patients who received the combination had higher complete antiemetic response rate (61% versus 46%) and less nausea as per visual analog scale (18% versus 33%) [20]. Fox et al. conducted a phase III trial in 44 patients comparing ondansetron with combination of ondansetron, dexamethasone, and chlorpromazine in multiday cisplatin chemotherapy. There was a reduction in total number of emetic episodes in favor of the combination arm but this did not reach statistical significance (55 versus 32%, *p* value = 0.22). Mean change of nausea control measured by visual analog scale (VAS) was superior with the combination regimen. It is unsure what role chlorpromazine played in this study and it is questionable that it added much value to the ondansetron and dexamethasone combination [21]. A bigger phase III trial demonstrated that adding dexamethasone to dolasetron significantly increased effectiveness in preventing nausea and vomiting related to fractionated cisplatin chemotherapy. Complete response rates were significantly better in all 5 days in the combination arm compared to dolasetron alone [22]. These studies clearly established the superiority of 5-HT_3_ receptor antagonist-dexamethasone combination in multiday cisplatin based chemotherapy and soon became the standard of care.

However optimal duration of dexamethasone has never been clearly established. Side effects of 5 consecutive days of dexamethasone during cisplatin administration and three additional days for delayed nausea and vomiting and repeated courses every 3 weeks are going to be substantial [26]. In a retrospective study evaluating dexamethasone toxicity in patients receiving moderately emetogenic chemotherapy, all patients received dexamethasone (10 or 20 mg intravenously) and oral dexamethasone for delayed prophylaxis (4 mg twice daily for 2-3 days). In this study, 45% of patients reported moderate to severe problems with insomnia, 27% had indigestion or epigastric discomfort, and 27% had agitation [27]. Late toxicity from dexamethasone is of particular concern, particularly with avascular necrosis of the hip on long term dexamethasone [26, 28]. Our antiemetic prophylaxis regimen for cisplatin chemotherapy in germ cell tumors restricts dexamethasone to days 1 and 2 of cisplatin administration (Table 2).

Palonosetron is a unique 5-HT_3_ receptor antagonist with activity at both central and GI sites and also has a long plasma terminal phase elimination half-life of approximately 40 hours [29]. A phase II trial by Einhorn et al. [23] evaluated palonosetron 0.25 mg IV on chemotherapy days 1, 3, and 5 plus dexamethasone in patients receiving multiday cisplatin chemotherapy for germ cell tumor. Majority of patients had no emesis at any time throughout days 1–5 (51%) or days 6–9 (83%), had no moderate or severe nausea, and did not require rescue medication. Most patients reported that the nausea they experienced had no significant effect on daily functioning. Recently, two studies have demonstrated that single dose (0.25 mg IV) of palonosetron maintains response rates for acute and delayed CINV over repeated courses of chemotherapy in patients on HEC [30, 31]. An open-label, single-arm, and multicenter study was performed in patients with testicular germ cell tumor who were scheduled to receive 5-day cisplatin based combination chemotherapy. The antiemetic therapy consisted of palonosetron 0.75 mg on day 1, aprepitant 125 mg on day 1, 80 mg on days 2 to 5, dexamethasone 9.9 mg on day 1, and 6.6 mg on days 2 to 8. Complete response (CR) rate, which was defined as no vomiting and no rescue medication use, was achieved in 90% of the patients in the first chemotherapy course, and high CR rates were also observed in the second and third courses (82.1 and 78.3%, resp.) [25]. This trial was conducted in Japan, where 0.75 mg intravenous dose of palonosetron is the commonly used dose. Trials evaluating single dose (0.25 mg intravenously) of palonosetron or head to head comparison of palonosetron compared to ondansetron in multiday cisplatin chemotherapy have not been performed. Our antiemetic prophylaxis recommends palonosetron 0.25 mg IV on days 1, 3, and 5, based on the phase II trial results. Ondansetron 16 mg orally daily on days 1–5 can be alternatively used in patients who are unable to get palonosetron due to insurance reasons or other issues (Table 2).

## 5. NK**_**1**_** Receptor Antagonists

Introduction of NK_1_ receptor antagonists substantially increased our ability to control nausea and vomiting and is now an important component of antiemetic management strategies in multiday cisplatin based chemotherapy. In the first multicenter, double-blind, and placebo-controlled trial conducted by Navari et al., addition of NK_1_ receptor antagonists (aprepitant) to granisetron and dexamethasone resulted in significant decrease in acute CINV and delayed emesis for single day HEC [32]. In 3 studies comparing the addition of aprepitant (125 mg on day 1, 80 mg on days 2 and 3) to intravenous ondansetron 32 mg (on day 1) and oral dexamethasone (12 or 20 mg on days 1 and 8 or 16 mg on days 2 and 3), addition of aprepitant resulted in absolute increase in complete response rate from 11% to 20%, favoring aprepitant. Also there was a significant benefit observed in prevention of delayed emesis [33–35].

Albany et al. (see Table 1) conducted the first randomized, double-blind, placebo-controlled, and phase III crossover study evaluating the oral NK_1_ antagonist aprepitant in combination with several 5-HT_3_ receptor antagonists (except palonosetron) and dexamethasone in patients with germ cell tumors receiving 5-day cisplatin combination chemotherapy [24]. Patients were randomly assigned to aprepitant 125 mg on day 3 and 80 mg per day on days 4 through 7 or to placebo with initial course and crossover to opposite treatment with the second course. Among 69 patients in the study, 42% achieved complete response for days 1–8, defined as no emetic episodes with no use of rescue medication with aprepitant, compared with 13% in the placebo group (*p* value < 0.01). Only 16.2% of patients who got aprepitant had one or more emetic episodes compared to 47.1% with placebo. No statistically significant difference was noted in visual analog scale (VAS) for nausea, but it was numerically superior with aprepitant. Also patient preference was higher for aprepitant cycle compared to placebo. Dexamethasone was used only on days 1 and 2 for the acute phase to decrease the adverse effects of corticosteroids. This may have contributed to the loss of control on days 3 through 5 during the acute phase and on days 6 through 8 in the delayed phase. Utilizing dexamethasone in patients receiving three to four cycles of BEP chemotherapy would put them at higher risk for multiple complications including insomnia, hyperglycemia, and avascular necrosis of hip. Palonosetron, with its longer half-life, is probably more attractive in this setting and is included in our antiemetic prophylaxis regimen (Table 2).

Pharmacokinetics interactions of aprepitant should be carefully evaluated in clinical practice. It has been noted to decrease plasma concentrations of other agents that are metabolized by CYP3A4 or CYP2C9 [36]. Aprepitant has been shown to cause a twofold increase in the area under the plasma concentration curve (AUC) of dexamethasone, which is a sensitive substrate of CYP3A4 and therefore oral steroid dose should be reduced by 50% when used in combination with aprepitant. There might also be interaction with warfarin and it would require close monitoring [37].

Fosaprepitant is a water-soluble phosphoryl prodrug for aprepitant which is administered intravenously and rapidly converts to aprepitant within 30 minutes of administration [38]. Single dose fosaprepitant (150 mg intravenously) in combination with granisetron 40 *μ*g/kg intravenously on day 1 and dexamethasone on days 1–3 was evaluated in a phase II trial against control regimen of placebo plus intravenous granisetron and dexamethasone. Complete response (no emesis and no rescue therapy) was significantly higher in the fosaprepitant group than in control group (64% versus 47%, resp.). The fosaprepitant regimen was more effective than the control regimen in both the acute phase (94% versus 81%, *p* = 0.0006) and the delayed phase (65% versus 49%, *p* = 0.0025) [39]. Currently, the Hoosier Clinical Research Network (HCRN) is conducting a multicenter, phase II study of fosaprepitant with 5-HT_3_ receptor antagonists and dexamethasone in patients with germ cell tumors undergoing 5-day cisplatin based chemotherapy.

## 6. Novel Agents and Emerging Therapies

Netupitant and palonosetron (NEPA) is the first antiemetic combination agent developed, comprised of a new, highly selective NK_1_ receptor antagonist, netupitant, and the 5-HT_3_ receptor antagonist, palonosetron. Hesketh et al. conducted a phase II randomized double-blind study in 694 patients undergoing cisplatin based chemotherapy with combination of NEPA. Different oral doses of netupitant (100, 200, and 300 mg) and palonosetron 0.50 mg, all given on day 1, were compared to a standard 3-day regimen of aprepitant, with ondansetron as an additional exploratory arm. All NEPA doses showed superior overall complete response rates compared with palonosetron (87.4%, 87.6%, and 89.6% for NEPA100, NEPA200, and NEPA300, resp., versus 76.5% palonosetron; *p* value < 0.050) with the highest NEPA300 dose studied showing an incremental benefit over lower NEPA doses for all efficacy endpoints. NEPA showed superiority for all key efficacy end points of no emesis, no significant nausea, and complete protection (no emesis and no significant nausea) rates even during the delayed (25–120 h) phase. Safety profile was similar to aprepitant and ondansetron [40]. This trial specifically excluded patients getting multiday cisplatin chemotherapy but appears to be a promising therapy warranting further evaluation with randomized clinical trials in multiday cisplatin chemotherapy.

Olanzapine is an atypical antipsychotic medication which blocks multiple neurotransmitter receptors including dopaminergic, serotonergic, adrenergic, and histamine receptors. Two phase III clinical trials have been conducted to evaluate efficacy and safety of olanzapine compared with 5-HT_3_ receptor antagonists. In a study by Tan et al., 229 patients receiving moderately emetogenic chemotherapy (MEC) and highly emetogenic chemotherapy (HEC) were randomly assigned to azasetron, dexamethasone, and olanzapine or to azasetron and dexamethasone. Complete response rate in HEC group was significantly improved for both the delayed period (69.64% versus 30.43%, *p* < 0.05) and the overall period (69.64% versus 28.26%, *p* < 0.05), but no difference was noted in the acute phase. Patients reported better quality of life on olanzapine [41]. Navari et al. conducted an additional phase III study in patients receiving HEC. 249 patients were randomized to either olanzapine or aprepitant in combination with palonosetron and dexamethasone. Rate of control of chemotherapy induced emesis was comparable between the two regimens while nausea was significantly better controlled with olanzapine arm particularly in the delayed phase [42]. Both these studies did include cisplatin based chemotherapy but excluded multiday cisplatin chemotherapy. So it is difficult to draw any conclusions about the effectiveness of olanzapine in patients undergoing multiday cisplatin chemotherapy without further trials. Other newer agents like rolapitant and gabapentin have also not yet been studied in multiday chemotherapy setting.

## 7. Practice Guidelines and Our Practice

Practice guidelines (from the National Comprehensive Cancer Network (NCCN), Multinational Association of Supportive Care in Cancer/European Society for Medical Oncology (MASCC/ESMO), and American Society of Clinical Oncology (ASCO)) recommend a combination of antiemetic agents for the prevention of CINV with HEC, specifically “triple therapy” with an NK_1_ receptor agonist, a 5-HT_3_ receptor agonist, and dexamethasone [43–45], to be given prophylactically on all days of chemotherapy regimen.

At Indiana University, our protocol for antiemetic prophylaxis for multiday cisplatin combination chemotherapy utilizes all three classes of recommended treatments and is detailed in Table 2.

## 8. Conclusion

5-HT_3_ receptor antagonists, NK_1_ receptor antagonist aprepitant, and dexamethasone have substantially improved our ability to prevent and control acute and delayed nausea and vomiting in multiday cisplatin based chemotherapy. In spite of significant progress made, the challenge remains in achieving complete control as any deviation in chemotherapy treatment course or schedule due to CINV in this curable cancer is unacceptable. Mechanism and pattern of CINV with multiday chemotherapy and efficacy of antiemetic drugs may differ from those observed in single day chemotherapy. Newer prophylaxis regimens with fosaprepitant, NEPA, and olanzapine have demonstrated excellent results with control of acute and delayed nausea and vomiting in HEC, but these have not been validated in multiday regimens yet. Rigorous examination of newer agents with randomized clinical trials with special emphasis on multiday chemotherapy is essential to determine the efficacy, optimal dose, and duration of these agents and combinations.

## Figures and Tables

**Table 1:** Selected phases II and III trials of various agents for treatment of chemotherapy induced nausea and vomiting in patients undergoing multiday cisplatin based chemotherapy.

Study	Phase	*N*	CINV prophylaxis	Emesis		Nausea
				Day 1 CR	Days 1–5 CR	
Einhorn et al., 1990 [14]	II	36	Ondansetron	77%	30%	NA

Sledge et al., 1992 [15]	III	45	Ondansetron versus metoclopramide	78% versus 14% (*p* < 0.001)	30% versus 9% (*p* = 0.002)	VAS scores 8 versus 58.5 (*p* < 0.001)

Bremer, 1992 [17]	III	200	Granisetron versus alizapride (substituted benzamide) + dexamethasone	90% versus 66% (*p* < 0.001)	49% versus 35%.	NA

Fox et al., 1993 [21]	III	45	Ondansetron + dexamethasone + chlorpromazine versus ondansetron	95% versus 82%	55% versus 32% (*p* = 0.22)	VAS scores 5.5 versus 15 (*p* = 0.046)

Noble et al., 1994 [18]	III	200	Ondansetron versus granisetron	NA	40% versus 44% Patient preference, 34% versus 26% (*p* = 0.048)	NA

Fauser et al., 2000 [22]	III	210	Dolasetron + dexamethasone versus dolasetron	NA	73% versus 41% (*p* < 0.0001). CR rates on each study day were also significantly higher (*p* = 0.029)	VAS score of 0 (no nausea) day 1: 88% versus 60% (*p* < 0.001); day 5: 63% versus 37% (*p* = 0.017)

Einhorn et al., 2007 [23]	II	41	Palonosetron + dexamethasone	88%	51%	No or mild nausea, self-reported 59%

Albany et al., 2012 [24]	III	69	Aprepitant + 5-HT_3_ RA (other than palonosetron) + dexamethasone versus aprepitant + dexamethasone	NA	47% versus 15% (*p* = 0.01)	VAS: aprepitant better than placebo on all 6 days

Hamada et al., 2014 [25]	II	30	Aprepitant + palonosetron + dexamethasone	NA	90%	Mild to no nausea 70%

CR: complete response (no emesis and no need for rescue medication); VAS: visual analog scale for nausea; 5-HT_3_ RA: 5-hydroxytryptamine receptor antagonists; NA: not available.

**Table 2:** Antiemetic prophylaxis regimen for multiday cisplatin chemotherapy for germ cell tumors, Indiana University protocol.

Day 1	Day 2	Day 3	Day 4	Day 5	Days 6–8
Dexamethasone 20 mg IV	Dexamethasone 20 mg IV	Palonosetron 0.25 mg IV		Palonosetron 0.25 mg IV	Dexamethasone 4 mg BID PO

Palonosetron^*∗*^ 0.25 mg IV		Fosaprepitant 150 mg IV		Fosaprepitant 150 mg IV	

^*∗*^Alternatively, ondansetron 16 mg orally daily can be utilized from days 1–5 if palonosetron is not available.
